# A Novel Predictive Nomogram including Serum Lipoprotein a Level for Nonsentinel Lymph Node Metastases in Chinese Breast Cancer Patients with Positive Sentinel Lymph Node Metastases

**DOI:** 10.1155/2021/7879508

**Published:** 2021-11-22

**Authors:** Zeng Fang, Ruizhi Wang, Ciqiu Yang, Dong Wang, Wanna Chen, Bo Lin, Dongsheng Gong, Songqi Li, Jiadong Liang, Xiaoli Liang, Chunxian Zeng, Jie Li, Kun Wang, Weiming Lv

**Affiliations:** ^1^Department of Breast and Thyroid Surgery, First Affiliated Hospital, Sun Yat-sen University, China; ^2^Department of Laboratory Medicine, First Affiliated Hospital, Sun Yat-sen University, China; ^3^Department of Breast Surgery, Guangdong Provincial People's Hospital, Guangdong Academy of Medical Science, China; ^4^Shenzhen Key Laboratory of Viral Oncology, Clinical Innovation & Research Center, Shenzhen Hospital, Southern Medical University, China

## Abstract

**Background:**

We developed a new nomogram combining serum biomarkers with clinicopathological features to improve the accuracy of prediction of nonsentinel lymph node (SLN) metastases in Chinese breast cancer patients.

**Methods:**

We enrolled 209 patients with breast cancer who underwent SLN biopsy and axillary lymph node dissection. We evaluated the relationships between non-SLN metastases and clinicopathologic features, as well as preoperative routine tests of blood indexes, tumor markers, and serum lipids, including lipoprotein a (Lp(a)). Risk factors for non-SLN metastases were identified by logistic regression analysis. The nomogram was created using the R program to predict the risk of non-SLN metastases in the training set. Receiver operating characteristic (ROC) analysis was applied to assess the predictive value of the nomogram model in the validation set.

**Results:**

Lp(a) was significantly associated with non-SLN metastasis status. Compared with the MSKCC model, the predictive ability of our new nomogram that combined Lp(a) level and clinical variables (pathologic tumor size, lymphovascular invasion, multifocality, and positive/negative SLN numbers) was significantly greater (AUC: 0.732, 95% CI: 0.643–0.821) (C-index: 0.703, 95% CI: 0.656–0.791) in the training cohorts and also performed well in the validation cohorts (C-index: 0.773, 95% CI: 0.681–0.865). Moreover, the new nomogram with Lp(a) improved the accuracy (12.10%) of identification of patients with non-SLN metastases (NRI: 0.121; 95% CI: 0.081–0.202; *P* = 0.011).

**Conclusions:**

This novel nomogram based on preoperative serum indexes combined with clinicopathologic features facilitates accurate prediction of risk of non-SLN metastases in Chinese patients with breast cancer.

## 1. Introduction

The sentinel lymph node (SLN) is the first organ reached by metastasizing cancer cells. At present, the SLN biopsy procedure is used to assess regional lymph node involvement in patients with breast cancer. The standard treatment for breast cancer patients with SLN metastases is complete axillary lymph node dissection (ALND) [1, 2]. However, approximately 50% of patients with positive SLN do not have additional nodal metastases after ALND [3]. Therefore, many patients undergo unnecessary ALND, with no additional therapeutic benefit or further staging information provided. Thus, the need for complete ALND in patients with SLN metastases and at low risk of lymph node metastases is controversial [4, 5].

New models for accurate prediction of the status of non-SLN metastasis in patients with metastasis-positive SLN and to distinguish patients with low risk of lymph node metastasis are urgently required to avoid unnecessary ALND. Currently, a prediction model designed by the Memorial Sloan Kettering Cancer Center (MSKCC) is widely used in clinical practice [6]. This model is user-friendly and provides precise and individualized estimate advantages; however, it has been reported that the MSKCC nomogram, which is based on samples from Western countries (reported AUC: 0.77), is unsatisfactory for prediction in Chinese populations (tested AUC: from 0.683 to 0.700) [7, 8].

Previous studies and MSKCC models have suggested that clinicopathologic features, such as pathologic size, lymphovascular invasion, multifocality, and positive/negative SLN numbers, are independent risk factors for non-SLN metastases [6–8]. However, the correlation between preoperative blood indexes and non-SLN metastases in patients with positive SLN biopsies remains to be determined. Furthermore, no predictive nomogram for the risk of non-SLN metastases in patients with a positive SLN biopsy based on clinicopathologic features and preoperative serum markers has yet been developed. Therefore, in this study, we aimed to identify some markers in blood as risk factors for non-SLN metastases and to construct a novel nomogram combined with MSKCC model factors and blood markers for the prediction of non-SLN metastases in Chinese patients with positive SLN metastasis to guide treatment.

## 2. Materials and Methods

### 2.1. Patients

This retrospective study was approved by the Ethics Committee of the First Affiliated Hospital, Sun Yat-sen University and Guangdong Provincial People's Hospital (China). Data were retrieved from the medical records of patients who underwent surgery for breast cancer at the First Affiliated Hospital, Sun Yat-sen University and Guangdong Provincial People's Hospital. Patient records were anonymized and deidentified prior to analysis, and the requirement to obtain written informed consent from patients was waived by the committee approval because of the retrospective study design.

A total of 209 patients with metastasis-positive SLNs who underwent ALND between January 2014 and July 2019 were retrospectively enrolled in this study. The following inclusion criteria were applied: (1) histologically confirmed primary breast cancer before surgery, (2) no preoperative distant metastases, and (3) clinicopathologic features and data for serum markers including routine blood test indexes (white blood cell (WBC), Neu (neutrophil), Lym (lymphocyte), platelet (PLT), neutrophil to lymphocyte ratio (NLR), and platelet to lymphocyte ratio (PLR)), tumor markers (carcinoembryonic antigen (CEA), carbohydrate antigen 153 (CA153), and carbohydrate antigen 125 (CA125)), and serum lipids (cholesterol (Chol), triglyceride (TG), low-density lipoprotein cholesterol (LDL-c), high-density lipoprotein cholesterol (HDL-c), apolipoprotein A (ApoA), apolipoprotein B (ApoB), apolipoprotein E (ApoE), and Lp(a)) were clearly recorded. The exclusion criteria were as follows: (1) prior history of SLN biopsy, (2) any preoperative chemotherapy or radiotherapy, (3) with another malignancy or life-threatening disease diagnosed during the three years prior to surgery, and (4) death in hospital due to postoperative complications. Of the enrolled patients, 116 were assigned to the training set and 93 were assigned to the validation set.

### 2.2. Identification of Combined Serum Markers and Clinicopathologic Features

Preoperative data for serum markers, including routine blood indexes (WBC, Neu, Lym, PLT, NLR, and PLR), tumor markers (CEA, CA153, and CA125), and lipids (Chol, TG, LDL-c, HDL-c, ApoA, ApoB, ApoE, and Lp(a)) were standardized by measurement using the same tests and reported using consistent units. The clinicopathologic features of age, pathologic tumor size, tumor type (ductal vs. lobular), lymphovascular invasion (yes vs. no), multifocality (yes vs. no), estrogen-receptor status (yes vs. no), histological grade (I/II vs. III), and positive and negative SLN numbers were analyzed. The presence and absence of SLN metastases were diagnosed histologically and defined as non-SLN(+) and non-SLN(-), respectively.

### 2.3. Statistical Analysis and Nomogram Construction

All statistical analyses were performed using SPSS version 19.0 and R (version3.1.2 URL http://www.R-project.org/). Unordered categorical variables were analyzed using the Chi-squared test. Logistic regression analysis was used to analyze risk factors for non-SLN metastases. A nomogram was used as a model to evaluate the value of factors for predicting the risk of non-SLN metastases in patients. The predictive accuracy of the nomogram was then validated by ROC analysis and quantified by calculation of the area under the ROC curve (AUC). An AUC of 0.5 indicates no relationship while an AUC of 1.0 indicates perfect concordance. Moreover, the nomogram was subjected to 1,000 bootstrap resamples for reduction of overfit bias and for internal validation with a logistic calibration plot. A two-sided *P* value < 0.05 was considered to indicate statistical significance. Net reclassification improvement (NRI) was determined using R software to evaluate the improvement of the nomogram compared with the MSKCC nomogram.

## 3. Results

### 3.1. Clinical Characteristics

In total, 209 breast cancer patients who underwent ALND were enrolled in this study. The average age of the patients was 41 years (range, 21–79 years). Of these patients, 116 (58 non-SLN(-) and 58 non-SLN(+))were allocated to the training cohort and 93 (41 non-SLN(-) and 52 non-SLN(+)) were allocated to the validation cohort. The baseline clinical and pathological characteristics of all the study participants are listed in Table 1. The baseline clinicopathologic factors were similar in the two cohorts. The rates of non-SLN(+) were 50.0% and 55.9% in the training and validation cohorts, respectively.

### 3.2. Nomogram Based on Clinical Factors from the MSKCC Model and Predictive Ability of the MSKCC Model in Chinese Patients

**Figure 1:**
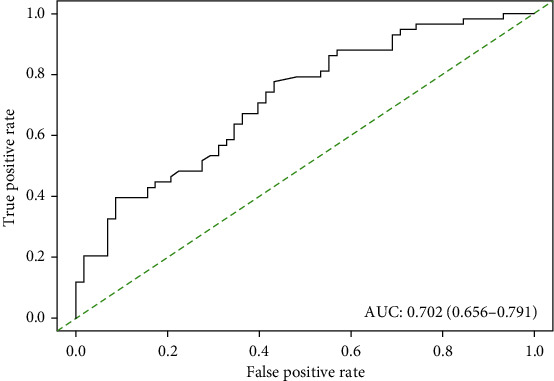
A receiver operating characteristic (ROC) curve of the MSKCC model illustrated an AUC of 0.702 (95% CI: 0.656-0.791), which revealed a worse ability to estimate the status of non-SLN metastasis in Chinese patients.

Using the MSKCC model, a score was assigned to each patient in the training cohort. The total score calculated using the nomogram corresponded to a predictive value for the risk of non-SLN metastasis. A ROC curve was generated to validate the predictive accuracy of the nomogram; the AUC was 0.702 (95% CI: 0.656–0.791) (Figure 1).

**Figure 2:**
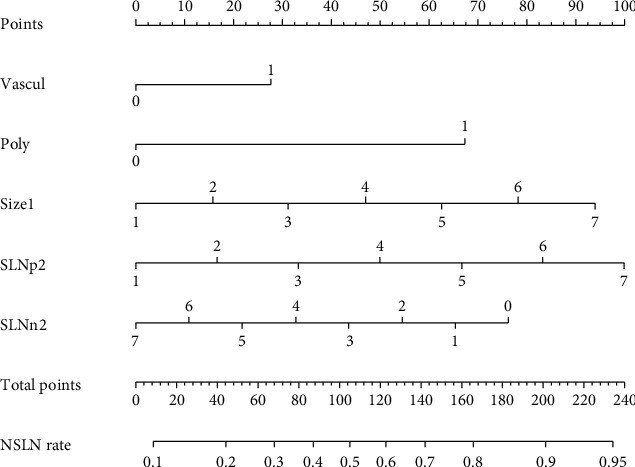
A nomogram composed of clinical-related factors from the MSKCC model in primary cohort. The risk value of lymph node metastasis was calculated by drawing a vertical line to the point on the axis for each of the factors. The points for each factor were summed and located on the total point line. And then, the bottom line corresponding vertically to the above total line illustrated the individual predictive value for lymph node metastasis.

**Figure 3:**
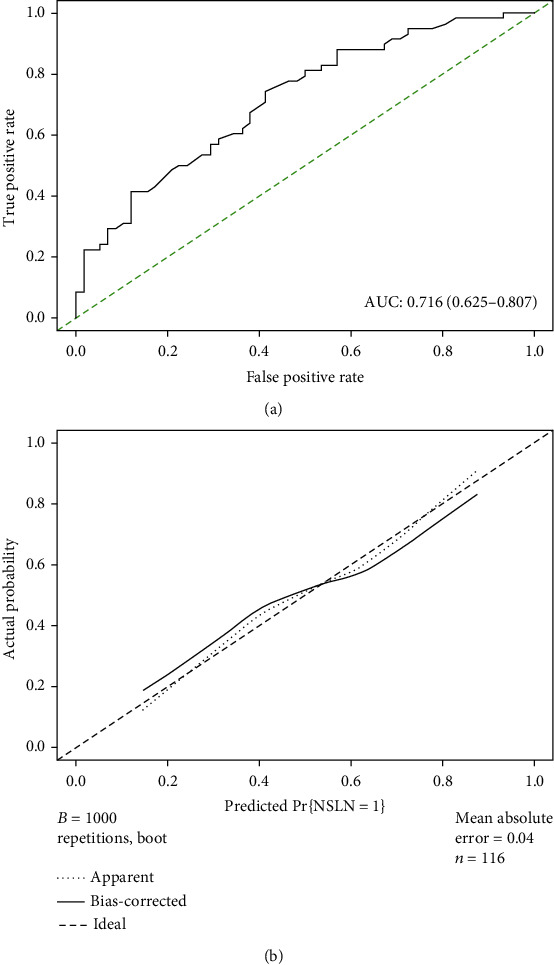
ROC curve and calibration plot of nomogram in primary cohort. (a) ROC curve of the model illustrated an AUC of 0.716 (95% CI: 0.625-0.807), which revealed a better ability to estimate the status of non-SLN metastasis in Chinese patients. (b) Calibration plot of a nomogram.

We then constructed a nomogram based on the clinical factors from the MSKCC model to predict the risk of non-SLN metastasis for patients in the training primary cohort (Figure 2). A ROC curve and calibration plot were generated to validate the predictive accuracy of this nomogram model (Figure 3). The AUC of the ROC curve for this model was 0.716 (95% CI: 0.625–0.807).

These results confirmed the inferior ability of the nomogram for predicting the status of lymph node involvement in Chinese patients compared with previous reports in Western patients (reported AUC: 0.77) [6].

### 3.3. Identification of Risk Factors and Multivariate Analysis for Prediction of Risk of Non-SLN Metastasis

Logistic regression analysis was performed to determine the risk factors for non-SLN metastasis (Table 2). In the univariate analysis, clinicopathologic features, such as age, pathologic tumor size, lymphovascular invasion, multifocality, estrogen-receptor status, numbers of positive and negative SLNs, CEA, CA125, LDL-c, HDL-c, and Lp(a), were found to be significantly associated with non-SLN metastasis. In the multivariate analysis, pathologic tumor size, numbers of positive and negative SLNs, and Lp(a) were identified as independent risk factors for non-SLN metastasis.

### 3.4. Nomogram Combining Lp(a) and Clinical-Related Factors from the MSKCC Model Predicts Non-SLN Metastasis

**Figure 4:**
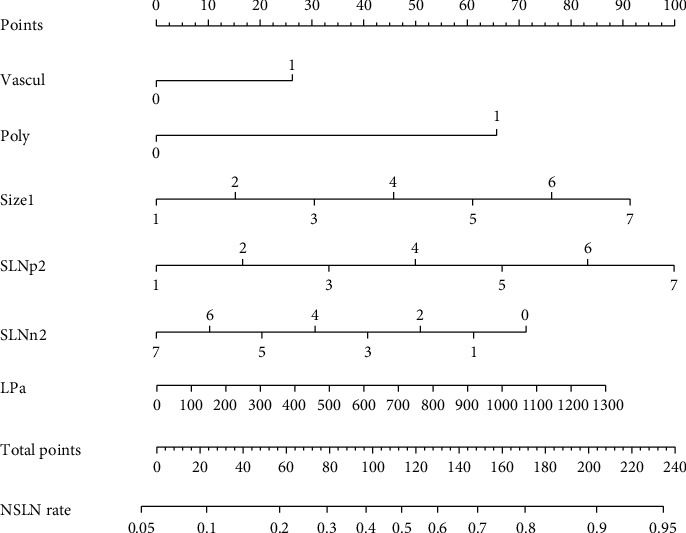
A nomogram composed of Lp(a) and clinical-related factors from the MSKCC model in primary cohort. Constructed Lp(a) nomogram to predict non-SLN metastasis for Chinese patients, with the pathologic size, tumor type (ductal vs. lobular), lymphovascular invasion (yes vs. no), multifocality (yes vs. no), estrogen-receptor status (yes vs. no), No. of positive SLN, No. of negative SLN, and Lp(a) level.

**Figure 5:**
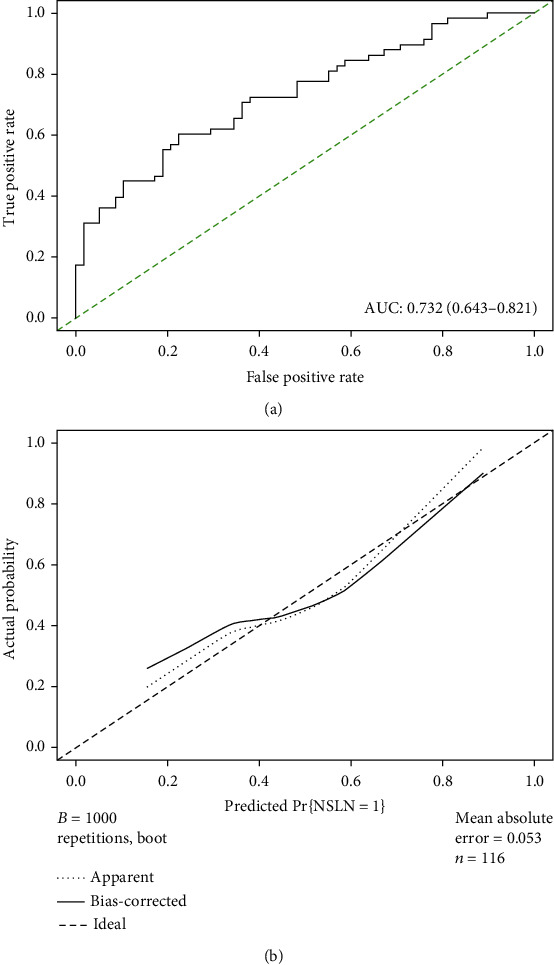
ROC curve and calibration plot of new nomogram in primary cohort. (a) ROC curve of the model illustrated an AUC of 0.732 (95% CI: 0.643-0.821), which revealed a good concordance and a reliable ability to estimate the status of non-SLN metastasis in Chinese patients. (b) Calibration plot of a new nomogram.

A new nomogram was constructed to predict the risk of non-SLN metastasis for patients based on the combination of Lp(a), a novel independent risk factor identified in the training set, and clinical factors from the MSKCC model (Figure 4). For each patient, points were assigned for each of the clinicopathologic risk factors. A total score was calculated from the nomogram to predict the risk of non-SLN metastasis. A ROC curve and calibration plot were generated to validate the predictive accuracy of this nomogram model (Figure 5). The AUC of the ROC curve for this model was 0.732 (95% CI: 0.643-0.821), which revealed good concordance and a reliable ability to estimate the status of lymph node involvement.

To assess the validity of this model and evaluate the level of improvement in accuracy gained by using the selected clinicopathologic features and biomarkers in this new nomogram, we calculated the NRI between the new nomogram with Lp(a) and the nomogram without Lp(a). A shown in Table 3, the new nomogram with Lp(a) provides a 12.10% improvement in the accuracy of the model for identification of patients with non-SLN metastasis compared with the old nomogram (NRI: 0.121; 95% CI: 0.081–0.202; *P* = 0.011).

**Figure 6:**
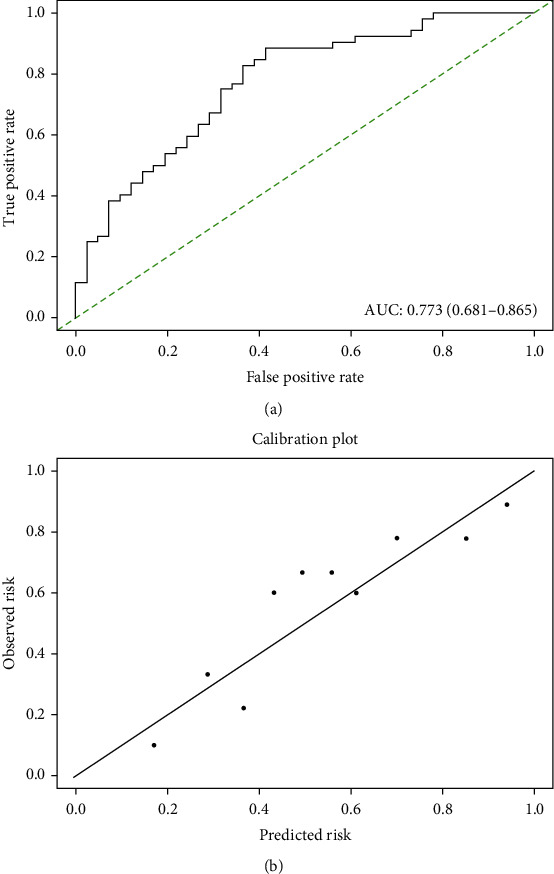
ROC curve and calibration plot of a new nomogram in validation cohort. (a) ROC curve of the model illustrated an AUC of 0.773 (95% CI: 0.681-0.865), which revealed a good concordance and a reliable ability to estimate the status of non-SLN metastasis in Chinese patients. (b) Calibration plot of a new nomogram.

Having developed a novel and promising nomogram model to predict the risk of non-SLN metastasis in patients with a positive SLN biopsy, we then examined its predictive ability in our validation cohort. The favorable calibration of the new nomogram was confirmed with the validation cohort, with a C-index of 0.773 (95% CI: 0.681-0.865) for the estimation of the risk of non-SLN metastasis (Figure 6).

## 4. Discussion

An effective method to improve the accuracy of predicting the risk of non-SLN metastasis in patients with a positive SLN biopsy is urgently needed in the era of precision medicine. In this study, we first analyzed common blood indexes to identify differential biomarkers in the training cohort and found that Lp(a) was found to be significantly associated with the risk of non-SLN metastasis. We next constructed a new nomogram based on the combination of clinicopathologic features from the MSKCC model and Lp(a) levels using the training cohort. The performance of this new nomogram for prediction of the risk of non-SLN metastasis was verified in the validation cohort. The subgroup of patients identified with a very low risk of non-SLN metastasis using this nomogram might be candidates for observation rather than immediate ALND, which is recommended for the patients identified with a high risk of non-SLN metastasis. Thus, our nomogram may improve the prediction of non-SLN metastasis status and guide individualized therapies for patients with a positive SLN biopsy.

Accumulating evidence shows a close relationship between the level of blood lipids and the occurrence and development of tumors, including breast cancer [9–13]. However, the mechanism underlying this correlation is not completely clear, and the data are inconsistent [14–18]. For example, in a study of 2,724 patients with breast cancer conducted in Sweden over a period of four years, Olsson reported a positive correlation with breast cancer in individuals with hypolipidemia and diabetes, which is inconsistent with the results obtained in studies in China [13]. Serum lipids represent a group of indicators with a variety of functions. In accordance with these characteristics, different, and even directly conflicting, conclusions on the relationships between these factors and breast cancer have been reported. Studies have shown that cholesterol metabolites (27-hydroxycholesterol) in humans have estrogenic functions and bind to estrogen receptors in breast tumor cells, thus promoting the proliferation of breast tumor cells [19]. These results highlight the potential of statins or dietary management to reduce the risk of the occurrence, progression, and recurrence of breast cancer. However, some studies have shown that cholesterol metabolites inhibit the occurrence and development of breast cancer. Dendrogenin A, a metabolite produced by cholesterol and histamine metabolism, has been shown to stimulate breast cancer cell redifferentiation both in vitro and in vivo [20]. In addition, new data have shown that the incidence, recurrence rate, and mortality of breast cancer are positively correlated with the intake of high-fat dairy products [21].

Lp(a) is a cholesterol-rich macromolecular lipoprotein [22] that exists at relatively stable concentrations in serum. LP(a) levels are determined mainly by genetic factors and are largely unaffected by statins, age, sex, smoking, diet, environment, lipid metabolism, and other factors [23–25]. This suggests the potential value of serum LP(a) as a predictive biomarker. Lp(a) is an independent risk factor for cardiovascular diseases such as coronary artery disease, peripheral vascular disease, and calcified aortic valve disease [26–30]. However, our study is the first to provide evidence of the relationship between Lp(a) and breast cancer.

Previous studies have evaluated the risk of non-SLN metastasis in patients with a positive SLN biopsy based on a combination of clinicopathologic features and a micro-RNA-based signature or one-step nucleic acid amplification [30, 31]. Although these studies showed high accuracy for the prediction in non-SLN metastasis, such approaches are inconvenient in clinical practice. Therefore, we evaluated the potential of blood biomarkers as an effective approach for the prediction of non-SLN metastasis in patients with a positive SLN biopsy. Our results suggest that the evaluation of serum Lp(a) levels could be an important tool for the management of patients with positive SLN, assisting in the stratification of patients that may harbor non-SLN metastases, aiding in therapy planning and patient staging, and ultimately contributing to an improvement in quality of life and survival rates.

Several limitations of our study should be noted. First, the results could be influenced by inherent biases and variation associated with a retrospective study design. Second, the model was established and validated in cohorts from the same ethnic group (Chinese). As breast cancer patients in China are often younger than in those in other countries, our prediction model may not be directly applicable in non-Asian patients. Fourth, only a relatively small number of patients were enrolled in our study; therefore, our results required confirmation in large-scale, multiethnic, and multicenter clinical trials.

## 5. Conclusions

In this study, we evaluated the value of serum biomarkers for accurate prediction of non-SLN metastasis in patients with a positive SLN biopsy and revealed an association between serum Lp(a) levels and lymph node metastases in breast cancer. Our results suggest that serum Lp(a) levels, which can be conveniently obtained prior to surgery, can be combined with clinicopathologic features (pathologic tumor size, lymphovascular invasion, multifocality, and positive and negative SLN numbers) to accurately predict the risk of non-SLN metastases. This novel nomogram model has the potential for convenient use to optimize current treatment strategies by avoiding unnecessary ALND procedures.

## Figures and Tables

**Table 1:** Descriptive characteristics of the primary cohort and validation cohort.

	Primary cohort (*n* = 116)		Validation cohort (*n* = 93)		*P*
	*n*	%	*n*	%	
Age (year)					0.607
≤50	66	56.9	49	52.7	
>50	50	43.1	44	47.3	
Pathologic size (cm)					0.103
≤0.5	7	6.0	2	2.2	
0.6-1.0	19	16.4	9	9.7	
1.1-2.0	59	50.9	29	31.2	
2.1-3.0	26	22.4	31	33.3	
3.1-4.0	2	1.7	15	16.1	
4.1-5.0	2	1.7	5	5.4	
≥5.1	1	0.9	2	2.1	
Tumor type					0.976
Ductal	114	98.3	92	98.9	
Lobular	2	1.7	1	1.1	
Lymphovascular invasion					0.339
No	94	81.0	81	87.1	
Yes	22	19.0	12	12.9	
Multifocality					0.900
No	112	96.6	88	94.6	
Yes	4	3.4	5	5.4	
Estrogen-receptor status					0.718
Negative	17	14.7	12	12.9	
Positive	99	85.3	81	87.1	
No. of positive SLN					0.255
1	57	49.1	48	51.6	
2	27	23.3	20	21.5	
3	16	13.8	13	14.0	
4	9	7.8	3	3.2	
5	4	3.4	4	4.3	
6	1	0.9	2	2.2	
7	2	1.7	3	3.3	
No. of negative SLN					0.314
0	20	17.2	14	15.1	
1	20	17.2	9	9.7	
2	26	22.4	18	19.4	
3	16	13.8	18	19.4	
4	13	11.2	16	17.2	
5	6	5.2	7	7.5	
6	9	7.8	5	7.2	
7	6	5.2	5	7.2	
Non-SLN metastases					0.397
Yes	58	50.0	52	55.9	
No	58	50.0	41	54.1	

**Table 2:** Univariable and multivariable logistic regression analysis of factors associated with the incidence of non-SLN metastases.

Clinical variables	*P*
Univariable analysis	
Age	0.072
Pathologic size	0.083
Tumor type (ductal vs. lobular)	0.999
Lymphovascular invasion (yes vs. no)	0.160
Multifocality (yes vs. no)	0.332
Estrogen-receptor status (yes vs. no)	0.195
Grade (I/II vs. III)	0.521
No. of positive SLN	0.015
No. of negative SLN	0.008
WBC	0.743
Neu	0.781
Lym	0.995
PLT	0.896
NLR	0.690
PLR	0.804
CEA	0.152
CA125	0.115
CA153	0.547
Chol	0.857
TG	0.404
LDL-c	0.325
HDL-c	0.303
ApoA	0.909
ApoB	0.628
ApoE	0.950
Lp(a)	0.130
Multivariable analysis	
No. of positive SLN	0.009
No. of negative SLN	0.009
Pathologic size	0.038
Lp(a)	0.028

**Table 3:** Improved predictive ability of new nomogram compared to the MSKCC model in Chinese patients by NRI.

	Improved ability (95% CI)	*P* value
NRI	0.121 (0.081-0.202)	0.011

## Data Availability

Data are available on request. Our data are available on request through contacting the corresponding author (lvwm@mail.sysu.edu.cn).

## Additional Points

*Highlights*. (i) Lp(a) is an independent risk factor for non-SLN metastases. (ii) We constructed a new nomogram combining clinicopathologic features and Lp(a). (iii) Our nomogram accurately predicted non-SLN metastasis in clinical practice
